# Longitudinal changes in home food availability across the first 3 years of life and associations with family context predictors

**DOI:** 10.3389/fnut.2023.1215894

**Published:** 2023-09-28

**Authors:** Barbara H. Fiese, Jennifer M. Barton, Esra Sahin

**Affiliations:** ^1^Family Resiliency Center, University of Illinois Urbana-Champaign, Urbana, IL, United States; ^2^Department of Human Development and Family Studies, University of Illinois Urbana-Champaign, Urbana, IL, United States

**Keywords:** childhood obesity, home food availability, family, childcare, STRONG Kids2

## Abstract

**Background:**

There is limited research tracking changes in home food availability during the first 3 years of life and whether the family context influences these changes.

**Objective:**

This study examined changes in and predictors of home food availability across the first 3 years of life.

**Design:**

This study utilized longitudinal data from the STRONG Kids2 birth cohort from the target child at 6 weeks to 36 months postpartum.

**Participants:**

Mothers of 468 children were surveyed at 6 weeks, 3, 12, 24, and 36 months postpartum.

**Methods:**

Home observations were completed by trained research assistants to complete the presence of foods in the home. The primary outcomes were the availability of 10 food groups and scores from the Home Food Inventory (HFI), including dairy (regular fat), dairy (reduced fat), processed meats, other meats and non-dairy protein, savory snacks, vegetables, vegetables (no potatoes), and three obesogenic scores. Repeated measures ANOVA were used to examine changes in the HFI food groups and obesogenic scores over time. Multilevel regressions were conducted to examine whether the presence of an older sibling, entry into childcare, and mother’s return to full-time work were associated with the HFI.

**Results:**

Significant changes were detected for dairy (regular fat), other meats and non-dairy protein, savory snacks, vegetables, vegetables (no potatoes), and all obesogenic scores across time. A linear trend occurred for most HFI groups, however, the third obesogenic score (without milk and cheese) was highest at 3 months, declined at 12 months, and then slowly increased from 12 to 36 months years. The presence of an older sibling was a consistent predictor of the HFI groups over time. Entry into childcare was only associated with the availability of processed meats.

**Conclusion:**

The availability of food types shift as children age and their dietary needs alter. It is important to consider the whole family context such as the presence of older siblings whose dietary needs may differ from younger children. Future efforts are warranted to consider changes in food availability among diverse samples and different family structures.

## Introduction

### Background/rationale

The first 3 years of life reflects rapid changes in diet and growth. From birth to 12 months of age the child transitions from either breast milk or formula (or a combination) to solid foods and the introduction of dairy products (1). From 12 to 24 months the toddler is exposed to an increased variety of foods including fresh fruits and vegetables and dietary guidelines recommend against providing infants and young children with sugar-sweetened beverages (1). From 24 to 36 months, the child develops more autonomy and expresses food preferences which may shape overall family food shopping habits. This is also the period in which picky eating behaviors are evident and families may adjust meals to conform to the child’s desires (2, 3).

One way to track changes in diet during the child’s early years is the availability of food in the home. Home food availability (e.g., fresh fruits and vegetables, sugar-sweetened beverages) has been found to be related to fruit and vegetable consumption in preschool age children (4), school age children (5), and adolescents (6). To date, these studies have been cross-sectional in nature, giving little indication on how there may be changes in home food availability across time. There have been a few intervention studies that indicate that home food availability is amenable to change (7, 8). However, the effect sizes are small and restricted to only a few food groups (e.g., number of fruits and vegetables, saturated fats). Therefore, there is a gap in the literature in terms of how the availability of different food groups change over time.

The family food environment may also be influenced by contextual variables beyond the age of the child. There are multiple socio-ecological factors that may influence home food availability. Several theoretical models have been proposed to account for variability in children’s diet including the Six 6 C’s model (9, 10), the ecologically informed model presented by Rosenkranz and Dzewaltowksi (11), and the ecological model proposed by Davison and Birch (12). What these theoretical models have in common is that there are multiple influences on child diet including culture, race, parental education, and the family context. To date, there have been few studies that have considered these socio-ecological factors in understanding variability in home food availability. There are some exceptions.

For example, Bauer and colleagues examined whether parental employment and work-family stress affected the family food environment (13). The investigators reasoned that working parents may spend less time cooking and shopping for food (14). The authors reported that maternal employment status affected the family food environment in those mothers who were employed full time were more likely to serve fast food at family meals and their children consumed fewer fresh fruits and vegetables. The first 3 years of life is an important developmental period in family life as parents make decisions about returning to work. Although there is considerable variability as to when mothers return to work after childbirth, those that return within 3 months after giving birth may place their children at nutritional risk (15), including increased weight gain (16).

Concomitant to a mother’s return to work is the child’s entry into childcare. Although the data is somewhat mixed as to whether participation in childcare raises a child’s risk for obesity, entry prior to 3 years of age appears to increase risk for overweight and obesity (17). It is not clear what the mechanism is for this effect as the provision of food is highly regulated in licensed childcare centers and homes. Children may receive up to three meals and two snacks in childcare settings. The Academy of Nutrition and Dietetics report that children who attend childcare full time receive one half to two-thirds of their daily nutrients at the center. In a report that examined the home-based diets of children who were enrolled in full time childcare, it was found that there was less consumption of fruits, vegetables and milk than recommended (18). Because this report was cross-sectional, it was not possible to determine whether these dietary patterns were evident prior to entry into childcare. Thus, considering mother’s return to work and entry into childcare and shifts in home food availability can address gaps in the literature.

There is very little research that considers home food availability and the presence or absence of siblings nor research that considers whether eating behaviors of young children are influenced by their siblings. Povey and colleagues identified sibling relationships as a potential barrier to healthy eating through teasing behaviors about eating fruits and vegetables and encouraging snacking behaviors (19). In an observational study of 27 singletons and 41 children with a sibling, the children without siblings scored lower on the Healthy Eating Index (HEI), including the consumption of empty calories (20). In a systematic review of peer and sibling influences on eating behaviors, none of the studies included children younger than 9 years of age or included the examination of food availability (21). In most home food availability studies, demographic variables such as household size is considered a control variable. The exception is a study by Amuto and colleagues who considered household occupancy as a central variable in demographic contributions to child fruit and vegetable consumption (22). None of the above-mentioned reports were longitudinal in design or included children younger than 3 years of age. Thus, we aimed to add to the literature to examine whether presence or absence of an older sibling influenced home food availability across the first 3 years of life. We reasoned that if there was the presence of an older sibling, there would be fewer changes in the availability of such foods as dairy when the child transitions to solid foods during the second year. However, if there were no older siblings in the household there may be more dramatic changes in the availability of different food types as the child is exposed to a variety of foods between 1 year and 3 years.

### Objectives

The objectives of this report are three-fold. First, we seek to identify changes in the availability of foods in the home over the first 3 years of life. We reason that because there are shifts in the child’s dietary needs that there should also be shifts in the presence of certain food types (e.g., dairy, fruits, and vegetables). We expected an increase in the presence of dairy products after the first year of life as well as an increase in the presence of fruits and vegetables.

Our second objective was to determine whether mother’s return to the work force and entry into childcare influenced home food availability. We reasoned that once mothers returned to work there would be added strains on time that may influence shopping patterns and result in a change in availability of foods. In addition, once the child enters into childcare and receives nearly half of their nutrients outside of the home, there may also be a change in the types of food available in the home. Because the literature is scant on these two topics, we consider this analysis exploratory.

Our third objective was to determine whether the presence or absence of an older sibling would influence home food availability. We reasoned that if there was the presence of an older sibling, there would be fewer changes in the availability of such foods as dairy when the child transitions to solid foods at and after 12 months of age. However, if there were no older siblings in the household there may be more dramatic changes in the availability of different food types as the child is exposed to a variety of foods between 12 and 36 months of age.

## Methods

### Study design

The STRONG Kids 2 (SK2) longitudinal, birth cohort panel study was designed to examine multilevel predictors of weight trajectories and dietary habits across the first 7 years of life. This study focuses on the surveys collected at 6 weeks and 3, 12, 24, and 36 months.

### Participants

Data for the current study were drawn from the SK2 birth cohort. Women were recruited from health care facilities and birthing classes during their third trimester of pregnancy from 2013 to 2017 in Central Illinois (for detailed description of the study protocol) (23). Exclusion criteria included premature birth (<37 weeks), birth conditions precluding normal feeding (e.g., cleft palate), and low birth weight (<2.50 kg). The final sample includes 468 mothers and their infants starting from 1-week postpartum. The initial sample size was determined by an *a priori* power analysis for the cohort study to detect weight trajectory differences to achieve 80% power to detect a difference of ≥27% between 2 child weight trajectory groups in a repeated measure design (23). The current study utilized both survey and home visit data beginning at 6 weeks and 3, 12, 24, and 36 months postpartum. Written informed consent was obtained from mothers at the initiation of the study and were instructed that they could withdraw at any time. Mothers were contacted via email or phone on their follow-up dates and a home visit was scheduled. The study was approved by the University of Illinois at Urbana-Champaign Institutional Review Board.

### Variables

#### Home food inventory

Home food availability was assessed with the Home Food Inventory (HFI) (24) at 3, 12, 24, 36 months. The HFI is a structured checklist of food and beverage items that may be available in the home; a trained research assistant completed this checklist with parents during a home visit. Scoring of the HFI results in 13 major food categories (some can be divided into regular fat and reduced fat), 2 ready access categories (healthy or unhealthy kitchen and refrigerator accessibility; e.g., ready to eat fruit, vegetables, savory snacks, soda), and an obesogenic score. All items on the HFI were scored as “Yes” (1 = item is available in home) and “No” (0 = item is not available in home). For the current study objectives, we used 7 categories/subscales: dairy (regular fat), dairy (reduced fat), processed meats, other meats and non-dairy protein, savory snacks, vegetables, and vegetables (no potatoes). We chose these categories as they are reflective of infant and toddler diet and most likely to change over time (as opposed to other categories that were less likely to be in an infant/toddler diet such as sweetened beverages). We also used the obesogenic score, which was calculated as the sum of regular fat cheese, regular fat milk, regular fat yogurt, regular fat other dairy, processed meat, regular fat frozen desserts frozen desserts, regular fat prepared desserts, high-sugar cereal, candy, and microwaveable or quick-cook food, as well as 22 individual items from added fats, savory snacks, beverages, unhealthy kitchen accessibility, and unhealthy refrigerator accessibility. Because regular fat dairy products are recommended for 12- and 24-month-old-children (25), we calculated two alternative obesogenic scores: one version that excludes regular fat milk and yogurt and one version that excludes both regular fat milk, yogurt, and cheese. The modified obesogenic scores were also calculated at 3 and 36 months to be used for comparison over time and with Fulkerson and colleagues’ original findings (24).

#### Family context predictors

Family context was characterized as entry into childcare and mother’s return to full-time work (by 3 months of age) and presence of an older sibling. At 3 months postpartum, mothers indicated whether their child had entered into childcare (1 = Yes, 0 = No) and whether they had to returned to full-time work at or by this time (1 ≤35 hours per week, 0  > 35 hours per week). At 6 weeks postpartum, mothers identified whether the focal child in the study was their first-born (0 = First-born) or if an older sibling was present in the home (1 = Older sibling).

### Statistical methods

#### Maternal and child covariates

Covariates included child biological sex, mother’s receipt of Women, Infants, and Children (WIC) benefits, education level, relationship status, mother’s age, and perceived social status. The covariates were selected based on the demographic variables selected for the SK2 birth cohort study described in the preliminary report (23). The following covariates were assessed at 6 weeks postpartum and were dummy coded: child biological sex (1 = Female, 0 = Male), mother’s receipt of WIC benefits (1 = Receipt of WIC, 0 = No receipt of WIC), maternal education (1 = College degree, 0 = Less than college degree), and maternal marital status (1 = Married, 0 = Not married). At 3 months postpartum, mother’s age was calculated and the single-item Perceived Social Status scale (26) was used to assess where mothers perceived their social status to be compared to others in their community, with “1” representing low perceived social status and “10” representing high perceived social status.

#### Statistical plan

First, descriptive statistics were examined for the 10 HFI scores of interest: dairy (regular fat), dairy (reduced fat), processed meats, other meats and non-dairy protein, savory snacks, vegetables, vegetables (without potatoes), obesogenic score (v1), obesogenic score (v2; without regular fat and yogurt), and obesogenic score (v3; without regular fat milk, yogurt, and cheese). Next, repeated measures ANOVAs were used to understand changes in the HFI scores across four time points: 3, 12, 24, and 36 months. A Bonferroni correction was applied to account for multiple *post hoc* pairwise comparisons. Last, multilevel modeling was used to account for the repeated measures nested within individuals over time (3, 12, 24, and 36 months). A series of multilevel regressions were conducted to examine whether entry into childcare, mother’s return to full-time work, and the presence of an older sibling were associated with the 10 HFI scores. In addition, the slope for each HFI score was estimated, and the correlation between each HFI score and its slope were controlled in each model. Maternal characteristics including receipt of WIC, age, perceived social status, education and relationship status, and child sex were included as covariates.

Data management, descriptive analyses, and repeated measures ANOVAs were conducted using Stata 17 (27), and the multilevel regressions were conducted using Mplus 8 (28). Given the longitudinal nature of the data, there is some missing data; missing data ranged from 3 to 18% over time, with the latter due to attrition at the later timepoints of data collection. However, the full information maximum likelihood (FIML) method in Mplus was used to allow for all cases to be included in estimation of the predictive models. The maximum likelihood robust (MLR) estimator was used to obtain robust standard errors. It can be difficult to ascertain power estimates for multilevel modeling (MLM) analyses because, to our knowledge, there is no widely accepted calculation of effect size in MLM. However, increasing the sample size at Level 2 (participants) may improve statistical power as opposed to increasing the sample size at Level 1 (time) (29, 30), with evidence from simulation studies indicating that a minimum of 10 groups are needed for multilevel modeling (31). The sample in the current study included up to 468 participants (Level 2) nested in 4 timepoints, which was sufficient to conduct multilevel regressions; the sample size of 468 families was determined to meet the target size required for the larger SK2 goals (23).

## Results

### Descriptive statistics

Table 1 provides descriptive statistics for the primary study variables. Nearly one-third of the sample reported the presence of an older sibling at 6 weeks of age, 42% of the children entered childcare by 3 months of age, and 62% of mothers had returned to full-time employment by the time their child was 3 months of age. On average, mothers had at least a Bachelor’s degree (74%) and were married (84%), with a smaller proportion of mothers receiving WIC benefits at 6 weeks (17%). The average age of mother’s was about 31 years, and their perceived social status was 6.16 out of a possible score of 10, and nearly half of the children in the sample were female (49%).

**Table 1:** Descriptive statistics of the family context predictors, demographic characteristics, and the Home Food Inventory.

	6 Weeks	3 Months	12 Months	24 Months	36 Months
	*n* (%)	*n* (%)/*M* (*SD*)	*M* (*SD*)	*M* (*SD*)	*M* (*SD*)
*n*	468	458	437	413	390
Family context predictors					
Presence of an older sibling	158 (34%)	–	–	–	–
Entry into childcare	–	188 (42%)	–	–	–
Mother return to full-time work	–	201 (62%)	–	–	–
Demographic characteristics					
Mother’s race/ethnicity					
Hispanic/Latino	19 (4%)	–	–	–	–
Non-Hispanic/Latino White	356 (76%)	–	–	–	–
Non-Hispanic/Latino Non-White	55 (12%)	–	–	–	–
African American	19 (4%)	–	–	–	–
Asian	30 (7%)	–	–	–	–
Alaskan Native or American Indian	1 (0.20%)	–	–	–	–
Unknown/missing	38 (8%)	–	–	–	–
Receipt of WIC	75 (17%)	–	–	–	–
Mother’s education: college	338 (74%)	–	–	–	–
Mother’s relationship: married	382 (84%)	–	–	–	–
Child biological sex: female	231 (49%)	–	–	–	–
Mother’s age	–	31.16 (4.51)	–	–	–
Perceived social status	–	6.16 (1.55)	–	–	–
Home Food Inventory scores					
Dairy	–	3.58 (1.66)	4.17 (1.71)	4.31 (1.67)	4.34 (1.75)
Dairy – reduced fat	–	2.89 (1.67)	3.03 (1.85)	3.13 (1.88)	3.17 (1.74)
Processed meats	–	1.56 (1.06)	1.56 (1.08)	1.60 (1.06)	1.66 (1.12)
Other meats and non-dairy protein	–	6.64 (1.63)	6.69 (1.62)	6.78 (1.60)	6.94 (1.69)
Savory snacks	–	5.27 (2.30)	5.36 (2.32)	6.40 (2.56)	6.62 (2.59)
Vegetables	–	9.56 (3.38)	10.66 (3.38)	10.41 (3.48)	10.63 (3.54)
Vegetables – no potatoes	–	8.89 (3.23)	9.86 (3.26)	9.64 (3.35)	9.90 (3.38)
Obesogenic score – v1	–	25.87 (7.69)	27.70 (7.86)	28.73 (7.63)	29.34 (7.71)
Obesogenic score – v2	–	25.87 (7.69)	25.89 (7.57)	26.87 (7.30)	27.54 (7.36)
Obesogenic score – v3	–	25.87 (7.69)	23.53 (6.97)	24.41 (6.82)	25.00 (6.92)

### Changes in the home food inventory across first 3 years of life

**Figure 1:**
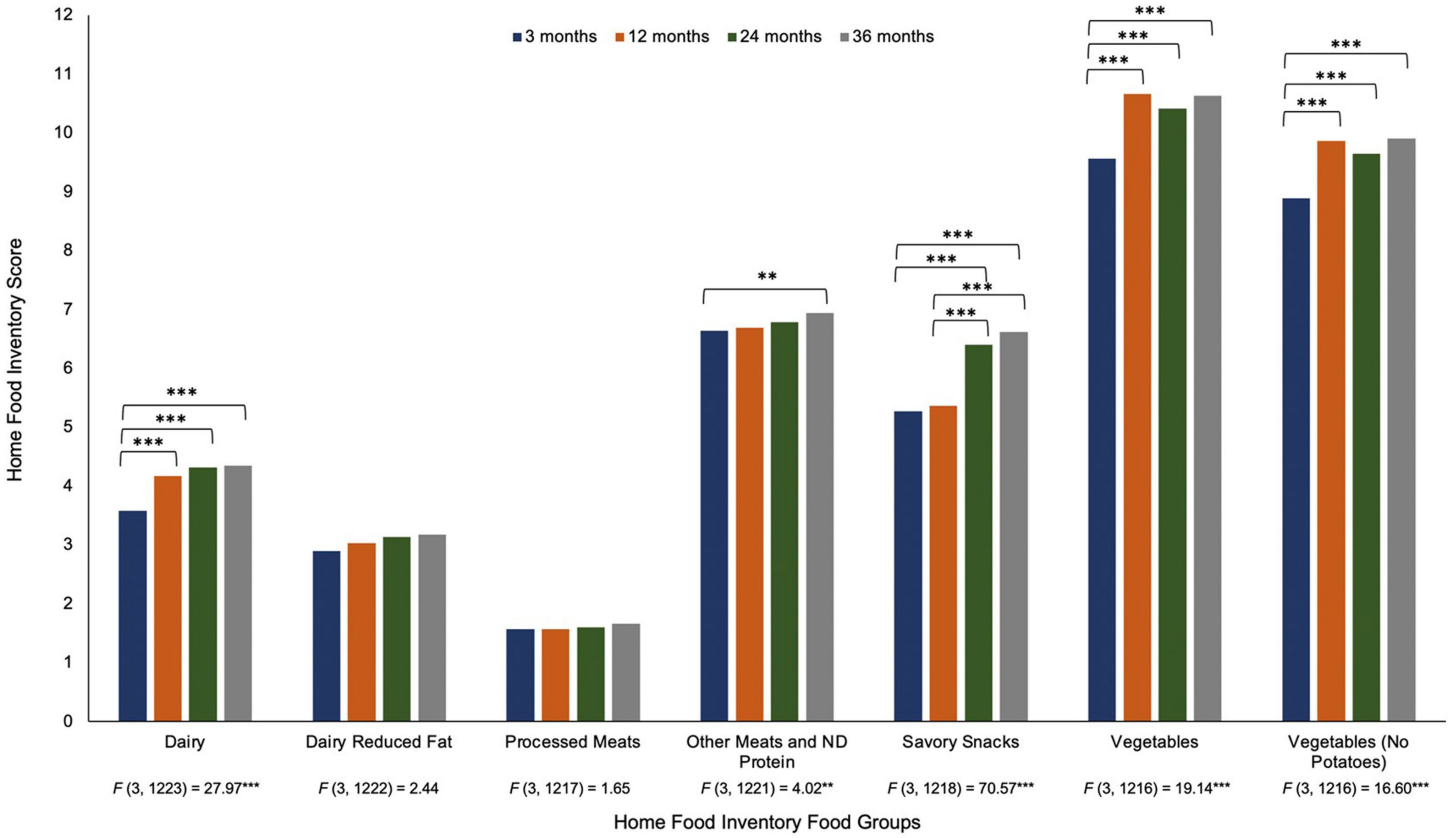
Repeated measures ANOVA of the Home Food Inventory group scores from 3 months to 36 months of age note. Changes in the Home Food Inventory groups across the first 3 years of life. Results of the repeated measures ANOVA of each HFI group across four time points are provided under the *x*-axis in the figure. *Post hoc* pairwise comparisons with a Bonferroni correction were used and presented above the bars in the figure. **p < 0.01, ***p < 0.001.

**Figure 2:**
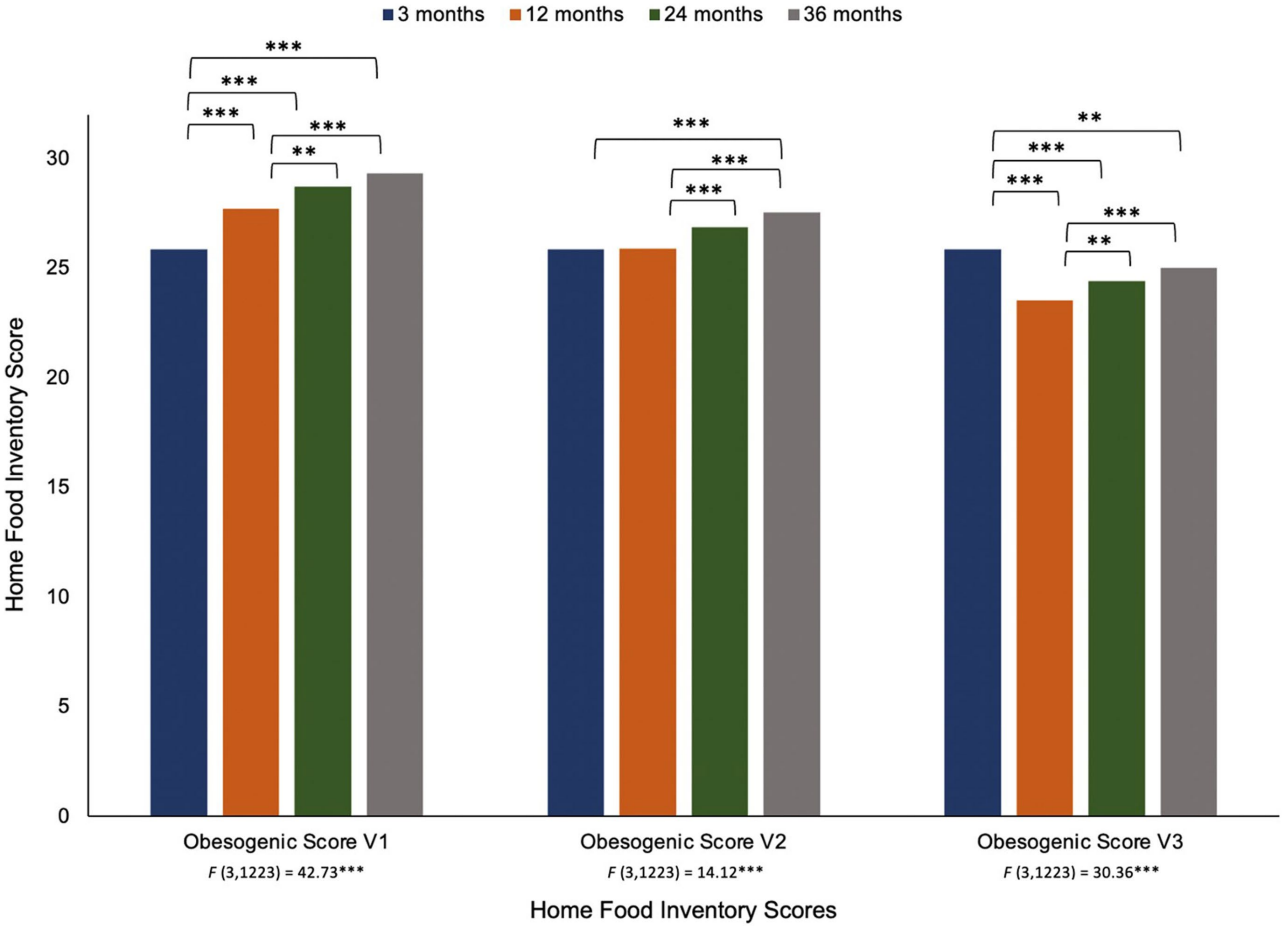
Repeated measures ANOVA of the Home Food Inventory obesogenic scores from 3 months to 36 months of age note. Changes in the Home Food Inventory groups across the first 3 years of life. Results of the repeated measures ANOVA of the HFI obesogenic scores across four time points are provided under the *x*-axis in the figure. *Post hoc* pairwise comparisons with a Bonferroni correction were used and presented above the bars in the figure. **p < 0.01, ***p < 0.001.

Figures 1, 2 present the changes in the HFI groups and obesogenic scores, respectively, across the first 3 years of life. Only the availability of dairy (reduced fat) and processed meats did not demonstrate significant differences over time. For dairy (regular fat) and both vegetable groups, 3 months was significantly lower than 12–36 months, but there were no significant changes between 12 and 36 months. Other meats and non-dairy protein at 3 months was only significantly lower than 36 months (no other differences were detected). Savory snacks increased between 12 and 24 months, with 3 and 12 months significantly lower than 24 and 36 months. We present the full results of the repeated measures ANOVAs with the pairwise comparisons tests (including *F*-tests and *t*-tests) in Supplementary Table 1.

**Table 2:** Associations between family context predictors and the Home Food Inventory (*n* = 468).

	Dairy		Dairy – reduced fat		Processed meats		Other meats and non-dairy protein		Savory snacks	
	B (SE)	*p*	B (SE)	*p*	B (SE)	*p*	B (SE)	*p*	B (SE)	*p*
Predictors										
Mother FTW 3M	0.05 (0.24)	0.831	−0.31 (0.27)	0.251	0.26 (0.15)	0.074	−0.12 (0.24)	0.602	−0.20 (0.33)	0.548
Childcare entry 3M	−0.01 (0.18)	0.962	0.30 (0.21)	0.148	**0.31 (0.12)**	**0.010**	−0.07 (0.20)	0.727	0.19 (0.25)	0.449
Older sibling	**0.85 (0.20)**	**<0.001**	0.23 (0.21)	0.273	**0.44 (0.13)**	**<0.001**	**0.58 (0.20)**	**0.003**	**0.90 (0.28)**	**0.001**
Covariates										
Receipt of WIC	−0.19 (0.29)	0.504	0.37 (0.32)	0.244	0.07 (0.19)	0.713	−0.06 (0.30)	0.833	**−0.86 (0.38)**	**0.023**
Education: college	0.10 (0.24)	0.678	**0.60 (0.24)**	**0.011**	−0.22 (0.16)	0.185	−0.35 (0.23)	0.122	0.44 (0.31)	0.163
Relationship: married	0.19 (0.27)	0.489	0.50 (0.28)	0.075	**−0.48 (0.20)**	**0.018**	0.26 (0.31)	0.399	0.11 (0.41)	0.778
Child sex: female	**−0.35 (0.18)**	**0.047**	−0.35 (0.19)	0.068	0.16 (0.11)	0.168	0.05 (0.03)	0.102	0.07 (0.24)	0.782
Mother’s age	−0.004 (0.02)	0.861	0.003 (0.02)	0.901	−0.02 (0.02)	0.386	0.05 (0.03)	0.102	−0.01 (0.03)	0.809
Perceived social status	−0.002 (0.07)	0.980	0.04 (0.07)	0.576	0.01 (0.04)	0.800	0.02 (0.07)	0.764	−0.15 (0.09)	0.100
Slope (*r*)	−0.007 (0.07)	0.929	−0.13 (0.11)	0.238	−0.05 (0.03)	0.119	**−0.20 (0.08)**	**0.009**	−0.04 (0.14)	0.760

	Vegetables		Vegetables – no potatoes		Obesogenic score – v1		Obesogenic score – v2		Obesogenic score – v3	
	B (SE)	*p*	B (SE)	*p*	B (SE)	*p*	B (SE)	*p*	B (SE)	*p*
Predictors										
Mother FTW 3M	0.44 (0.44)	0.315	−0.48 (0.42)	0.253	1.15 (1.09)	0.293	1.23 (1.08)	0.258	1.21 (1.06)	0.254
Childcare entry 3M	−0.21 (0.36)	0.555	−0.14 (0.35)	0.683	0.68 (0.84)	0.420	0.61 (0.84)	0.469	0.58 (0.83)	0.482
Older sibling	**1.15 (0.39)**	**0.004**	**1.00 (0.38)**	**0.009**	**5.09 (0.89)**	**<0.001**	**5.04 (0.89)**	**<0.001**	**5.13 (0.87)**	**<0.001**
Covariates										
Receipt of WIC	−0.01 (0.58)	0.987	0.16 (0.55)	0.770	**−2.57 (1.29)**	**0.045**	**−2.61 (1.27)**	**0.041**	**−2.62 (1.26)**	**0.037**
Education: college	0.91 (0.48)	0.056	**0.95 (0.45)**	**0.035**	−0.07 (1.08)	0.952	−0.16 (1.06)	0.882	−0.27 (1.04)	0.796
Relationship: married	0.42 (0.52)	0.417	0.44 (0.49)	0.370	−1.59 (1.44)	0.267	−1.62 (1.43)	0.256	−1.70 (1.42)	0.230
Child sex: female	0.29 (0.35)	0.399	0.28 (0.33)	0.398	−0.67 (0.79)	0.398	−0.67 (0.79)	0.396	−0.67 (0.78)	0.387
Mother’s age	**0.10 (0.05)**	**0.031**	**0.09 (0.05)**	**0.042**	−0.11 (0.13)	0.373	−0.11 (0.13)	0.372	−0.12 (0.13)	0.331
Perceived social status	0.12 (0.14)	0.386	0.12 (0.13)	0.363	−0.06 (0.31)	0.857	−0.03 (0.31)	0.916	0.01 (0.30)	0.988
Slope (*r*)	−0.13 (0.29)	0.650	−0.06 (0.26)	0.816	−1.92 (1.16)	0.096	**−2.77 (1.14)**	**0.015**	**−3.10 (1.16)**	**0.008**

Bolded estimates are statistically significant at *p* < 0.05 or less.

Mother return to FTW, mother returned to full time work by 3 months of age; Childcare entry 3M, child entered non-parental care by 3 months of age. The slope of each HFI score was estimated for each model, and the correlation between the slope and each HFI group was controlled for in each model.

The original obesogenic score (v1) increased from 3 to 36 months, with significant differences observed over time (except for between 24 and 36 months). However, when regular fat milk was removed from the obesogenic score (v2), the linear trend weakens such that 3 months only differs from 36 months, while 12 months differs from 24 and 36 months. In the third version of the obesogenic score, when both regular fat milk and cheese were removed, a new pattern of change was observed. Unlike the previous versions, the obesogenic score was now highest at 3 months and slowly increased from 12 to 36 months.

### Family context as predictors of the home food inventory across first 3 years of life

Supplementary Table 1 provides the associations between the family context predictors and the HFI outcomes. Entry into childcare by 3 months of age was only associated with increased availability of processed meats (*B* = 0.31, *p* = 0.010) over time. There was a marginal association observed between mother’s return to full-time work by 3 months and processed meats (*B* = 0.26, *p* = 0.074) availability. No additional significant associations between entry into childcare and mother’s return to full-time work were observed.

The presence of an older sibling was a consistent predictor of the HFI groups over time, except for dairy (reduced fat). Households with an older sibling had greater availability of regular fat dairy (*B* = 0.85, *p* < 0.001), processed meats (*B* = 0.44, *p* < 0.001), other meats and non-dairy protein (*B* = 0.58, *p* = 0.003), savory snacks (*B* = 0.90, *p* = 0.001), vegetables (*B* = 1.15, *p* = 0.004), and vegetables (no potatoes) (*B* = 5.09, *p* = 0.009), over time. In addition, households with an older sibling were also linked to greater obesogenic scores; obesogenic score – v1 (*B* = 5.09, *p* < 0.001), obesogenic score – v2 (*B* = 5.04, *p* < 0.001), and obesogenic score – v3 (*B* = 5.13, *p* < 0.001). Households with an older sibling were consistently characterized as more obesogenic over time. This pattern remained regardless of the version of obesogenic score, however, the mean scores slightly decreased when regular fat milk and cheese were removed from the scores.

The receipt of WIC was associated with lower availability of savory snacks (*B* = −0.86, *p* = 0.023) and lower obesogenic scores (all 3 versions) (*B*’s = −2.57 to −2.62, *p* < 0.05). Mother’s education was associated with greater availability of dairy (reduced fat) (*B* = 0.60, *p* = 0.011) and vegetables (no potatoes) (*B* = 0.95, *p* = 0.035), while processed meats (*B* = −0.48, *p* = 0.018) was less available in households when mothers were married. Mother’s age was associated with greater availability of vegetables (both with and without potatoes) (*B*’s = 0.09 to 0.10, *p* < 0.05), and dairy (regular fat) (*B* = −0.35, *p* = 0.047) was less available when the focal child was female. Significant, negative correlations between the slope and each outcome were observed for processed meats and two obesogenic scores (v2 and v3).

## Discussion

Our goal was to examine the longitudinal changes and contextual contributions to home food availability in families with a child between 3 and 36 months of age. Not surprisingly, we found increases in the presence of dairy (regular fat), savory snacks, and vegetables from the first year to the second and third years of life. The availability of other meats and non-dairy protein and dairy (reduced fat) were stable over time, while processed meats was relatively low and stable over time. This consistent increase in dairy availability corresponds to the dietary recommendations of supplementing human milk or infant formula with dairy milk (or alternatives) around 6 months of age and continuing with complementary feeding until about 24 months of age (25, 32). There was also an increase in the availability of vegetables and savory snacks at 24 and 36 months of age, which is consistent with the Feeding Infants and Toddlers Study (FITS) reporting low consumption of vegetables on a given day and increased consumption of bakery goods during the preschool years (33). Although the Home Food Inventory does not indicate dietary consumption patterns, the array of food available in the home corresponds to national reports on food consumption in the first 3 years of life.

We found partial support for family context factors as predictors of home food availability. Having an older sibling in the home resulted in greater availability of dairy, protein (processed and non-processed), savory snacks, vegetables, and obesogenic items across all time points. These findings reiterate the point that the presence of food in the home is a reflection of the family diet as a whole and is not driven by a single individual. There has been an effort to provide a broader view of the context in which home food availability data is collected. For example, Couch and colleagues considered the relation among the home food environment, feeding practices, parent and child characteristics, and child dietary intake (5). These researchers found that child diet quality was most closely associated with home food availability rather than feeding practices. This approach reflects a more contextual understanding of how availability of food in the home may be one of the multiple influences on child diet. We encourage future research to consider which aspects of the family context may affect the purchase and presence of different foods at different developmental periods.

A child’s entry into childcare nor a mother’s return to work by 3 months of age were strong predictors of home food availability. One reason for this null association may be that we did not assess other elements of the home food environment, including both the physical and sociocultural elements, that may have been more susceptible to being affected by a mother’s return to work. For example, Bauer and colleagues found that mothers and fathers who experienced high work-life stress also reported fewer family meals, spent less time preparing meals, and had less fruit and vegetable intake (13). In addition, we did not assess work-life stress which may affect the availability of more nutritious foods such as fruits and vegetables.

We created three different obesogenic scores in an attempt to be more developmentally sensitive and consistent with dietary recommendations. The obesogenic score proposed by Fulkerson and colleagues (24) included regular fat dairy products including milk and cheese. However, their sample included school-age children and adolescents, and thus, the proposed obesogenic score may not be appropriate for young children. The transition from human milk or formula to dairy products is recommended at 6 months of age and regular fat milk is recommended from 12 to 24 months. We considered three different scores: the original score that included all dairy products, a modified score that excluded regular fat milk, and a third modified score that excluded regular fat milk and cheese. These modifications weakened the linear effect across time and may more adequately reflect the contribution of dairy to a healthy diet between 12 and 36 months of life. There is considerable consensus that dairy intake is important for growth and development in early childhood and there is no association between milk intake by preschool children and concurrent or later childhood obesity (34).

Overall, there are several findings that researchers may consider when examining food availability in households with young children. First, the developmental context of food availability should be considered, for example, how the availability of certain foods may change as children age and their dietary needs change. This may be particularly important in the need to increase the availability of fresh fruits and vegetables and decrease the availability of snack foods and sugar sweetened beverages. Second, it is important to consider who is in the household because the availability of food items reflects the dietary preferences of the entire household. This notion is further supported by our finding that the availability of snack foods is higher when an older sibling is present. Although not examined in the current study, the food preferences of both parents and older siblings may explain the presence of various foods and beverages. For example, parents are likely to purchase and offers foods and beverages that they themselves prefer and enjoy (35, 36), and in turn, their children may be exposed to or develop a preference for similar foods and beverages (37). When constructing home based interventions, it is important to consider not only the target family member but also what the entire household pantry might hold.

This study is not without its limitations. Although we contribute to the literature by inclusion of longitudinal data, the sample under study is mainly white, highly educated, and married. It would be informative to include a more racially and economically diverse sample to see if the same patterns held. Because maternal reports of income in the current study sample were negatively skewed, we included their perceived social status as a covariate, however, no significant associations emerged. As recommended by Bruening and colleagues (38), future research would also benefit from considering a larger sample of families receiving federal food assistance such as SNAP and WIC. Although these researchers found some differences in home food availability of SNAP participants (particularly for less healthful foods), the sample was relatively small and the study design was cross-sectional. We found that participation in WIC was associated with lower presence of snacks and obesogenic scores. Participation in food programs such as SNAP and WIC need to be further explored in larger samples over time.

Of note, there is limited research using the HFI among households with toddlers (e.g., including 12 to 36 months) (39–41), however despite its use, the HFI has not been previously validated for use with infants or toddlers. The availability of healthy or unhealthy foods have been linked to consumption of fruits and vegetables or high-sugar/fat snack foods, respectively (40, 41). Cepni et al. (39) created a summary score of healthy food environment, including HFI items, and this was negatively related to maternal BMI (children ages 12–36 months). Similar reports using different measures of home food availability have found that the availability of fruit was related to infant fruit consumption (42) and home availability of fruits and vegetables was related to fruit and vegetable consumption in 18-month-olds (43). Although we cannot speak directly to the validity of the HFI for infants and toddlers across all food availability categories in this study, we do provide preliminary evidence that it is feasible and suggests patterns that are consistent with previous reports.

## Conclusion

To our knowledge, this is the first longitudinal study that examined changes in availability of food in the home across the first 3 years of life. Our findings suggest that availability of dairy, protein, vegetables, and snack foods increase over time and that these changes are affected by the family context such as the presence of an older sibling. Future studies are warranted to consider other contextual variables such as parental stress, family structure, and socio-economic status that may fluctuate and affect the family food environment.

## Data availability statement

The raw data supporting the conclusions of this article will be made available by the authors, without undue reservation.

## Ethics statement

The studies involving human participants were reviewed and approved by Institutional Review Board at the University of Illinois at Urbana-Champaign. Written informed consent to participate in this study was provided by the participating mothers.

## Author contributions

BF conceived the study, oversaw participant recruitment, oversaw data analysis, and contributed to the writing of the manuscript. JB contributed to study design, data analysis, and writing of the manuscript. ES contributed to study design, data analysis, and editing of the manuscript. All authors contributed to the article and approved the submitted version.

## Abbreviations

STRONG Kids 2: Synergistic Theory and Research on Nutrition Group Kids 2
HFI: Home Food Inventory
ANOVA: Analysis of Variance
HEI: Healthy Eating Index
WIC: Women Infants and Children program
FIML: Full Information Likelihood
MLR: Maximum Likelihood Robust
MLM: Multilevel Modeling
FITS: Feeding Infants and Toddlers Study
SNAP: Supplemental Nutrition Assistance Program
