# Supplementary material for: Longitudinal changes in home food availability across the first 3 years of life and associations with family context predictors
Source: Front Nutr. 2023 Sep 28;10:1215894. doi: 10.3389/fnut.2023.1215894 (PMC10569424; doi:10.3389/fnut.2023.1215894)
Supplement: Supplementary file 1 [file Table_1.docx]

| Supplemental Table 1  *Repeated measures ANOVA of the Home Food Inventory over time* | | | | | | | | | | |
| --- | --- | --- | --- | --- | --- | --- | --- | --- | --- | --- |
|  |  | |  | | Pairwise Comparisons (*t*-test) | | | | | |
| *Number of [items] in home* | | *F* | | *p* | 3M vs 12M | 3M vs 24M | 3M vs 36M | 12M vs 24M | 12M vs 36M | 24M vs 36M |
| Dairy | | **27.96** | | **< .001** | **6.23***** | **7.60***** | **7.97***** | 1.49 | 1.99 | .52 |
| Dairy – Reduced Fat | | 2.44 | | 0.06 | .93 | 2.14 | 2.36 | 1.24 | 1.47 | .25 |
| Processed Meats | | 1.65 | | 0.18 | .00 | .70 | 1.95 | .70 | 1.95 | 1.26 |
| Other Meats and ND Protein | | **4.02** | | **< .01** | .87 | 1.78 | **3.34**** | .93 | 2.49 | 1.58 |
| Savory Snacks | | **70.57** | | **< .001** | .15 | **9.49***** | **11.23***** | **9.35***** | **11.08***** | 1.91 |
| Vegetables | | **19.14** | | **< .001** | **6.87***** | **5.12***** | **5.94***** | -1.61 | -.66 | .91 |
| Vegetables – No Potatoes | | **16.60** | | **< .001** | **6.25***** | **4.66***** | **5.77***** | -1.46 | -.24 | 1.19 |
| Obesogenic Score – v1 | | **42.73** | | **< .001** | **5.07***** | **8.49***** | **10.54***** | **3.52**** | **5.67***** | 2.71 |
| Obesogenic Score – v2 | | **14.12** | | **< .001** | -.93 | 2.60 | **5.04***** | **3.51***** | **5.93***** | 2.48 |
| Obesogenic Score – v3 | | **30.36** | | **< .001** | **-9.28***** | **-5.76***** | **-3.39**** | **3.32**** | **5.51***** | 2.24 |
| *Note*. 3M = 3 months; 12M = 12 months; 24M = 24 months; and 36M = 36 months. ND = non-dairy protein.  ***p* < .01, ****p* < .001. | | | | | | | | | | |
